# 1,4-Bis(4-nitro­styr­yl)benzene

**DOI:** 10.1107/S1600536809024751

**Published:** 2009-07-11

**Authors:** Phuong-Truc T. Pham

**Affiliations:** a Department of Chemistry, Penn State Worthington Scranton, 120 Ridge View Drive, Dumore, Pennsylvania 18512, USA

## Abstract

The complete molecule of the title compound, C_22_H_16_N_2_O_4_, is generated by a crystallographic centre of inversion. The plane of the central aromatic ring is tilted by 11.85 (4)° with respect to the outer aromatic ring. The crystal packing is determined by van der Waals inter­actions, with stair-like stacking between adjacent aromatic rings. The stacks are staggered and each layer is approximately 3.8 Å from the next. The closest inter­molecular contact (approximately 2.42 Å) is between an O atom and a vinyl H atom.

## Related literature

For background information on photonic materials, see: He *et al.* (2008 ▶). For stilbenes, see: Moreno-Fuquen *et al.* (2008 ▶, 2009 ▶). For the synthesis, see: Borsche (1912 ▶); Nakatsuji *et al.* (1991 ▶). For a related structure, see: Bazan *et al.* (2000 ▶).
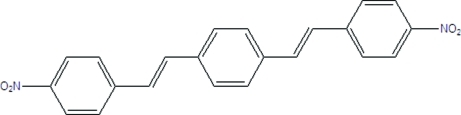


## Experimental

### 

#### Crystal data

C_22_H_16_N_2_O_4_
*M* *_r_* = 372.37
Monoclinic, 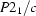

*a* = 7.4689 (12) Å
*b* = 16.615 (3) Å
*c* = 7.3917 (12) Å
β = 108.824 (3)°
*V* = 868.2 (2) Å^3^
*Z* = 2
Mo *K*α radiation
μ = 0.10 mm^−1^
*T* = 173 K
0.40 × 0.18 × 0.12 mm

#### Data collection

Bruker SMART Platform CCD diffractometer
Absorption correction: none
10088 measured reflections
2001 independent reflections
1486 reflections with *I* > 2σ(*I*)
*R*_int_ = 0.041

#### Refinement

*R*[*F* ^2^ > 2σ(*F* ^2^)] = 0.038
*wR*(*F* ^2^) = 0.116
*S* = 1.02
2001 reflections
159 parameters
All H-atom parameters refined
Δρ_max_ = 0.30 e Å^−3^
Δρ_min_ = −0.18 e Å^−3^

### 

Data collection: *SMART* (Bruker, 2001 ▶); cell refinement: *SAINT* (Bruker, 2001 ▶); data reduction: *SAINT*; program(s) used to solve structure: *SHELXS97* (Sheldrick, 2008 ▶); program(s) used to refine structure: *SHELXL97* (Sheldrick, 2008 ▶); molecular graphics: *SHELXTL* (Sheldrick, 2008 ▶); software used to prepare material for publication: *publCIF* (Westrip, 2009 ▶).

## Supplementary Material

Crystal structure: contains datablocks I, global. DOI: 10.1107/S1600536809024751/ng2592sup1.cif

Structure factors: contains datablocks I. DOI: 10.1107/S1600536809024751/ng2592Isup2.hkl

Additional supplementary materials:  crystallographic information; 3D view; checkCIF report

## Figures and Tables

**Fig. 1.**
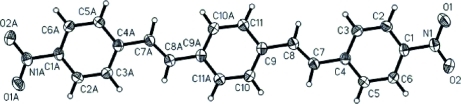
The molecular structure of 1,4-di(4-nitrostyryl)benzene with atom lables.

**Fig. 2.**
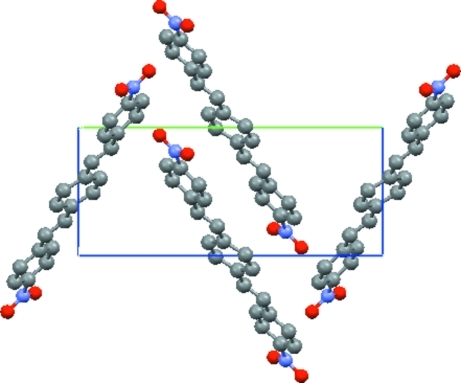
Crystal packing viewed along the a axis.

## supplementary crystallographic information

### Comment

Distyrylbenzene derivatives have been studied as laser dyes, components of organic light-emitting diodes, and as model compounds for the study of conductivity and molecular properties in substituted *p*-phenylenevinylene (PPV) polymers. For background information on photonic materials, see: He *et al.* (2008). For related systems of stilbene, see: Moreno-Fuquen *et al.* (2008, 2009). For literature related to the synthesis, see: Borsche (1912).

### Experimental

Synthesis was carried out following literature procedures (Nakatsuj) by standard Wittig synthesis. To a mixture of *p*-phenylenedimethylene- bis(tripheny1phosphonium chloride) (1.00 g, 1.43 mmol) and *p*-nitrobenzaldehyde (0.432 g 2.86 mmol) in EtOH (10 ml) was added 0.2 mol/*L* EtOLi(20 ml, 4.0 mmol) and the mixture was stirred overnight. The resulting reaction mixture was poured into water to give a yellow precipitate (0.4 g, 75%) which was filtered off, washed with EtOH, dried under reduced pressure, m.p. 289–290. Crystallization attempts from various solvents yielded only powders. Yellowish orange crystals however were grown by sublimation.

### Refinement

All hydrogen atoms were placed in ideal positions and refined as riding atoms with relative isotropic displacement parameters.

### Crystal data

**Table:**

C_22_H_16_N_2_O_4_	*F*(000) = 388
*M**_r_* = 372.37	*D*_x_ = 1.424 Mg m^−^^3^
Monoclinic, *P*2_1_/*c*	Mo *K*α radiation, λ = 0.71073 Å
Hall symbol: -P 2ybc	Cell parameters from 851 reflections
*a* = 7.4689 (12) Å	θ = 2.5–27.5°
*b* = 16.615 (3) Å	µ = 0.10 mm^−^^1^
*c* = 7.3917 (12) Å	*T* = 173 K
β = 108.824 (3)°	Needle, yellow
*V* = 868.2 (2) Å^3^	0.40 × 0.18 × 0.12 mm
*Z* = 2	

### Data collection

**Table:**

Bruker SMART Platform CCD diffractometer	1486 reflections with *I* > 2σ(*I*)
Radiation source: normal-focus sealed tube	*R*_int_ = 0.041
graphite	θ_max_ = 27.5°, θ_min_ = 2.5°
ω scans	*h* = −9→9
10088 measured reflections	*k* = −21→21
2001 independent reflections	*l* = −9→9

### Refinement

**Table:**

Refinement on *F*^2^	Primary atom site location: structure-invariant direct methods
Least-squares matrix: full	Secondary atom site location: difference Fourier map
*R*[*F*^2^ > 2σ(*F*^2^)] = 0.038	Hydrogen site location: inferred from neighbouring sites
*wR*(*F*^2^) = 0.116	All H-atom parameters refined
*S* = 1.01	*w* = 1/[σ^2^(*F*_o_^2^) + (0.0595*P*)^2^ + 0.1981*P*] where *P* = (*F*_o_^2^ + 2*F*_c_^2^)/3
2001 reflections	(Δ/σ)_max_ < 0.001
159 parameters	Δρ_max_ = 0.30 e Å^−^^3^
0 restraints	Δρ_min_ = −0.18 e Å^−^^3^

### Special details

**Table:**

Geometry. All e.s.d.'s (except the e.s.d. in the dihedral angle between two l.s. planes) are estimated using the full covariance matrix. The cell e.s.d.'s are taken into account individually in the estimation of e.s.d.'s in distances, angles and torsion angles; correlations between e.s.d.'s in cell parameters are only used when they are defined by crystal symmetry. An approximate (isotropic) treatment of cell e.s.d.'s is used for estimating e.s.d.'s involving l.s. planes.
Refinement. Refinement of *F*^2^ against ALL reflections. The weighted *R*-factor *wR* and goodness of fit *S* are based on *F*^2^, conventional *R*-factors *R* are based on *F*, with *F* set to zero for negative *F*^2^. The threshold expression of *F*^2^ > σ(*F*^2^) is used only for calculating *R*-factors(gt) *etc*. and is not relevant to the choice of reflections for refinement. *R*-factors based on *F*^2^ are statistically about twice as large as those based on *F*, and *R*- factors based on ALL data will be even larger.

### Fractional atomic coordinates and isotropic or equivalent isotropic displacement parameters (Å^2^)

**Table:**

	*x*	*y*	*z*	*U*_iso_*/*U*_eq_	
N1	−0.18351 (16)	0.18081 (7)	−0.31257 (17)	0.0314 (3)	
O1	−0.31485 (14)	0.14204 (7)	−0.29133 (16)	0.0424 (3)	
O2	−0.20374 (14)	0.23244 (7)	−0.43745 (15)	0.0415 (3)	
C1	0.00888 (18)	0.16483 (8)	−0.18509 (19)	0.0268 (3)	
C2	0.03516 (19)	0.10424 (8)	−0.0511 (2)	0.0293 (3)	
H2	−0.068 (2)	0.0758 (9)	−0.037 (2)	0.034 (4)*	
C3	0.2169 (2)	0.08605 (8)	0.0623 (2)	0.0299 (3)	
H3	0.234 (2)	0.0439 (10)	0.154 (2)	0.036 (4)*	
C4	0.37281 (19)	0.12795 (8)	0.04412 (18)	0.0274 (3)	
C5	0.3394 (2)	0.19080 (9)	−0.0888 (2)	0.0309 (3)	
H5	0.440 (2)	0.2209 (9)	−0.102 (2)	0.032 (4)*	
C6	0.1573 (2)	0.20926 (9)	−0.2043 (2)	0.0302 (3)	
H6	0.137 (2)	0.2519 (10)	−0.294 (2)	0.037 (4)*	
C7	0.56766 (19)	0.10807 (9)	0.1593 (2)	0.0304 (3)	
H7	0.659 (2)	0.1468 (9)	0.145 (2)	0.036 (4)*	
C8	0.62118 (19)	0.04369 (9)	0.27155 (19)	0.0296 (3)	
H8	0.530 (2)	0.0058 (9)	0.282 (2)	0.027 (4)*	
C9	0.81506 (18)	0.02312 (8)	0.38677 (18)	0.0270 (3)	
C10	0.9717 (2)	0.07130 (9)	0.39725 (19)	0.0291 (3)	
H10	0.959 (2)	0.1202 (10)	0.333 (2)	0.040 (4)*	
C11	0.8479 (2)	−0.04871 (9)	0.49188 (19)	0.0295 (3)	
H11	0.740 (2)	−0.0827 (10)	0.482 (2)	0.039 (4)*	

### Atomic displacement parameters (Å^2^)

**Table:**

	*U*^11^	*U*^22^	*U*^33^	*U*^12^	*U*^13^	*U*^23^
N1	0.0255 (6)	0.0340 (7)	0.0330 (6)	0.0040 (5)	0.0072 (5)	−0.0046 (5)
O1	0.0245 (5)	0.0545 (7)	0.0467 (7)	−0.0036 (5)	0.0091 (5)	−0.0009 (5)
O2	0.0348 (6)	0.0417 (6)	0.0426 (6)	0.0100 (5)	0.0050 (5)	0.0105 (5)
C1	0.0224 (6)	0.0296 (7)	0.0267 (7)	0.0041 (5)	0.0054 (5)	−0.0038 (5)
C2	0.0261 (7)	0.0292 (7)	0.0330 (7)	−0.0030 (5)	0.0103 (6)	−0.0011 (6)
C3	0.0309 (7)	0.0280 (7)	0.0299 (7)	−0.0001 (5)	0.0087 (6)	0.0026 (6)
C4	0.0267 (7)	0.0279 (7)	0.0260 (7)	0.0015 (5)	0.0064 (5)	−0.0017 (5)
C5	0.0246 (7)	0.0322 (7)	0.0355 (8)	−0.0021 (5)	0.0093 (6)	0.0031 (6)
C6	0.0295 (7)	0.0293 (7)	0.0312 (7)	0.0027 (5)	0.0089 (6)	0.0056 (6)
C7	0.0244 (7)	0.0331 (8)	0.0314 (7)	−0.0010 (6)	0.0059 (6)	−0.0005 (6)
C8	0.0260 (7)	0.0312 (7)	0.0300 (7)	0.0002 (6)	0.0071 (5)	−0.0017 (6)
C9	0.0278 (7)	0.0301 (7)	0.0221 (6)	0.0037 (5)	0.0066 (5)	−0.0032 (5)
C10	0.0313 (7)	0.0283 (7)	0.0268 (7)	0.0034 (5)	0.0082 (5)	0.0026 (5)
C11	0.0270 (7)	0.0313 (7)	0.0294 (7)	−0.0008 (5)	0.0082 (5)	−0.0011 (6)

### Geometric parameters (Å, °)

**Table:**

N1—O1	1.2253 (16)	C5—H5	0.932 (16)
N1—O2	1.2332 (15)	C6—H6	0.950 (16)
N1—C1	1.4661 (17)	C7—C8	1.334 (2)
C1—C6	1.3765 (19)	C7—H7	0.969 (16)
C1—C2	1.381 (2)	C8—C9	1.4640 (19)
C2—C3	1.379 (2)	C8—H8	0.951 (15)
C2—H2	0.940 (16)	C9—C10	1.399 (2)
C3—C4	1.4001 (19)	C9—C11	1.4020 (19)
C3—H3	0.953 (16)	C10—C11^i^	1.384 (2)
C4—C5	1.3999 (19)	C10—H10	0.930 (17)
C4—C7	1.4670 (19)	C11—C10^i^	1.384 (2)
C5—C6	1.3868 (19)	C11—H11	0.968 (17)
			
O1—N1—O2	123.49 (12)	C1—C6—C5	118.71 (13)
O1—N1—C1	118.79 (12)	C1—C6—H6	121.2 (10)
O2—N1—C1	117.71 (11)	C5—C6—H6	120.1 (10)
C6—C1—C2	122.13 (12)	C8—C7—C4	125.71 (13)
C6—C1—N1	119.44 (12)	C8—C7—H7	121.2 (9)
C2—C1—N1	118.42 (12)	C4—C7—H7	113.1 (9)
C3—C2—C1	118.68 (13)	C7—C8—C9	126.21 (14)
C3—C2—H2	120.4 (9)	C7—C8—H8	120.2 (9)
C1—C2—H2	120.9 (9)	C9—C8—H8	113.6 (9)
C2—C3—C4	121.26 (13)	C10—C9—C11	117.57 (12)
C2—C3—H3	118.2 (9)	C10—C9—C8	123.46 (13)
C4—C3—H3	120.6 (9)	C11—C9—C8	118.97 (13)
C5—C4—C3	118.20 (12)	C11^i^—C10—C9	120.98 (13)
C5—C4—C7	119.61 (13)	C11^i^—C10—H10	117.5 (10)
C3—C4—C7	122.18 (13)	C9—C10—H10	121.5 (10)
C6—C5—C4	120.96 (13)	C10^i^—C11—C9	121.45 (13)
C6—C5—H5	118.6 (9)	C10^i^—C11—H11	121.0 (9)
C4—C5—H5	120.5 (9)	C9—C11—H11	117.6 (9)
			
O1—N1—C1—C6	−178.44 (12)	N1—C1—C6—C5	−176.92 (12)
O2—N1—C1—C6	2.28 (19)	C4—C5—C6—C1	0.4 (2)
O1—N1—C1—C2	2.67 (19)	C5—C4—C7—C8	−170.57 (14)
O2—N1—C1—C2	−176.61 (12)	C3—C4—C7—C8	9.5 (2)
C6—C1—C2—C3	−2.3 (2)	C4—C7—C8—C9	179.87 (13)
N1—C1—C2—C3	176.57 (12)	C7—C8—C9—C10	1.9 (2)
C1—C2—C3—C4	0.3 (2)	C7—C8—C9—C11	−177.62 (13)
C2—C3—C4—C5	1.9 (2)	C11—C9—C10—C11^i^	−0.2 (2)
C2—C3—C4—C7	−178.18 (13)	C8—C9—C10—C11^i^	−179.77 (13)
C3—C4—C5—C6	−2.3 (2)	C10—C9—C11—C10^i^	0.2 (2)
C7—C4—C5—C6	177.80 (13)	C8—C9—C11—C10^i^	179.80 (13)
C2—C1—C6—C5	1.9 (2)		

Symmetry codes: (i) −*x*+2, −*y*, −*z*+1.
